# Resolving the Antarctic contribution to sea-level rise: a hierarchical modelling framework†

**DOI:** 10.1002/env.2247

**Published:** 2013-12-26

**Authors:** Andrew Zammit-Mangion, Jonathan Rougier, Jonathan Bamber, Nana Schön

**Affiliations:** a School of Geographical Sciences, University of BristolBristol, BS8 1SS, UK; b Department of Mathematics, University of BristolBristol, BS8 1TW, UK

**Keywords:** spatial statistics, stochastic partial differential equations, sea-level rise, source separation, remote sensing

## Abstract

Determining the Antarctic contribution to sea-level rise from observational data is a complex problem. The number of physical processes involved (such as ice dynamics and surface climate) exceeds the number of observables, some of which have very poor spatial definition. This has led, in general, to solutions that utilise strong prior assumptions or physically based deterministic models to simplify the problem. Here, we present a new approach for estimating the Antarctic contribution, which only incorporates descriptive aspects of the physically based models in the analysis and in a statistical manner. By combining physical insights with modern spatial statistical modelling techniques, we are able to provide probability distributions on all processes deemed to play a role in both the observed data and the contribution to sea-level rise. Specifically, we use stochastic partial differential equations and their relation to geostatistical fields to capture our physical understanding and employ a Gaussian Markov random field approach for efficient computation. The method, an instantiation of Bayesian hierarchical modelling, naturally incorporates uncertainty in order to reveal credible intervals on all estimated quantities. The estimated sea-level rise contribution using this approach corroborates those found using a statistically independent method. © 2013 The Authors. *Environmetrics* Published by John Wiley & Sons, Ltd.

## 1. INTRODUCTION

Larger than the conterminus USA, Antarctica is the biggest fresh water reservoir on Earth by an order of magnitude. It is estimated to contain 70% of the world's fresh water and if it were to melt, sea level would rise by about 60 m. As a consequence, even a relatively small change in the mass balance (the sum of ice gains and losses) could have profound implications for low-lying land. Sea-level rise is considered to be one of the most serious consequences of future climate warming, with over 185 million people living in coastal areas vulnerable to a 1 m increase (Nicholls, 2011). In the last two decades, the rate of sea level has risen to nearly twice the mean value for the 20th century, and part of that increase is believed to be due to contributions from Greenland and Antarctica, with the latter having the potential for the largest future contribution and the greatest uncertainty surrounding its predicted behaviour (Bamber and Aspinall, 2013).

The Antarctic contribution to sea-level rise within a time interval is proportional to its change in ice sheet mass within that same interval. This is a straightforward relationship and is the basis of all (including the present) analyses carried out so far. However, estimating the ice sheet mass loss/gain from available data is extremely challenging. First, we need to consider several physical processes with markedly different spatio-temporal characteristics, such as flow of the Earth's mantle, ice dynamics and precipitation. These are not directly observed as the observational data are a short recording of a *linear combination* of changes in height and mass caused by each of these processes. Thus, although only a subset of the processes are of direct relevance to sea-level rise, all must be considered. The problem of extracting individual components from observed linear combinations bears similarity to audio *source separation* (Cardoso, 1998), where sources composing a signal are separated from observed mixtures—we return to this discussion in Section 7.1. The second challenge regarding estimation is the presence of more latent components than observational data sets. Hence, the problem is, from a deterministic perspective, under-determined: even if we had noiseless data available at every desirable location, a unique solution for the latent variables does not exist.

In addition to challenges with regard to estimation, modelling the changes of the individual components is itself not straightforward. First, most processes are highly heterogeneous in nature. Precipitation, for instance, is considerably more variable at the coast than in the ice sheet interior. Second, some of the processes are highly correlated, that is, there is a linear dependence between a subset of these, which needs to be modelled. Third, the effect of these on the observed data is *spatially variant*. For example, the Antarctic ice sheet is surrounded by floating ice shelves that can exceed 1 km in thickness and 800 km in length and which are mainly in hydrostatic equilibrium with the ocean. If melted, these would not affect sea level nor the Earth's gravity field. Thus, whilst thinning of ice resting on bedrock results in a mass change, which is observed using satellite gravimetry, thinning of floating ice is not detected by gravimetry, although height changes are observed. The spatial dependence needs to be accurately captured for reliable estimation of ice sheet mass loss.

In addition to challenges concerning modelling and process separability, the sheer size of the problem requires special attention. Antarctica has an area of around 14 million km ^2^. Yet, significant ice loss occurs in regions with areas of only a few hundred km ^2^ (e.g. Rignot *et al.*, 2008). Modelling at these scales requires a high-dimensional latent space for accurate reconstruction. Data available from satellite remote sensing are today of sufficiently high resolution to resolve the detail required. However, the joint high dimensionality of the observation vector and latent space may quickly render inference computationally intractable. A further complication is the availability of a data set with a very large spatial footprint (satellite gravimetry data have a Nyquist rate of several hundred kilometres). We thus also need to consolidate the fine-scale modelling with the coarse scale of some of the available observations. This scale-mismatch, commonly referred to as *change of support* (Wikle and Berliner, 2005; Gelfand *et al.* 2001), has the undesirable effect of generating highly correlated posterior estimates. As a result, exact inference, even in the Gaussian case, is unscalable.

In related studies, all issues discussed previously are dealt with by fixing one or more of the unobserved physical processes to those obtained using physically derived climate/solid-earth numerical models (from now on, simply referred to as *numerical models*), see Section 2. This approach, however, brings with it the possibility of systematic error inclusion because of numerical model mismatch. The model mismatch, which is largely unknown, renders uncertainty quantification notoriously difficult. The aim of this work is to provide an alternative approach that avoids direct use of numerical models and attempts to tackle all aforementioned issues probabilistically. We do so by constructing a Bayesian hierarchical model (BHM) comprising an *observation model* (top layer), a *process model* (middle layer) and a *parameter model* (bottom layer) (see Berliner *et al.*, 2000; Cressie and Wikle, 2011, for further details and examples).

A BHM is ideally suited for carrying out inference on the Antarctic contribution to sea-level rise. First, the problems concerning estimation are catered for through the use of prior information, which reflect our prior understanding in the process model. A key novelty in this work is the way this is carried out—by extracting *characteristics* of available numerical models (such as length scales and process interdependencies) widely deemed to be representative of the true behaviour, for use as prior information. The procedure is shown in Section 3.1. Second, the modelling challenges are approached in a BHM by employing a class of spatial models frequently employed in spatial statistics (Cressie, 1993) and cross-covariance matrix functions (Finley *et al.* 2007; Gneiting *et al.* 2010), which are able to capture inter-process dependence. The observation model is then physically constructed with respect to the problem under study by considering how the processes generate the observed data in Section 4. This observation process relationship is an explicit link, which allows for powerful description (for example spatial heterogeneity/error characteristics) within the observation models. Third, the computational issues are solved in two stages: (i) by exploiting a recent result in spatial BHMs (Lindgren *et al.* 2011), which allows us to carry out approximations to the process model to generate sparse matrices amenable for fast linear algebraic computations; and (ii) by using a blocking strategy (Roberts and Sahu, 2002) motivated by the physical characteristics of the problem prior to Gibbs sampling. The latter implementation allows for fast computation, despite loss of sparsity in the update step caused by large spatial footprints of the observations.

The inferential advantages of employing a BHM are shown in Section 6 where we discuss the results, seen to corroborate those of other studies. Results are followed by a discussion on the adopted approach and a conclusion describing possible extensions in Section 7.

## 2. THE ANTARCTIC ICE SHEET: PHYSICAL PROCESSES AND CURRENT APPROACHES

The two key processes affecting the Antarctic ice sheet mass and thickness are (i) surface processes, such as precipitation and ablation (sublimation and melt-water runoff), and (ii) ice dynamics due to internal deformation of the ice due to gravity and sliding over a lubricated bed. The latter is influenced by changes in the force balance at the ice-ocean interface, termed the grounding line, and longer-term climate variations. A shift from the balance state (i.e. the state required for zero net mass loss) in either of these processes alters the ice sheet thickness and mass. The changes are detected by satellite altimetry (height change) and gravimetry (mass change). However, there are at least three other mechanisms influencing altimetry and gravimetry in addition to surface and ice dynamics, which need to be accounted for, despite not directly affecting ice sheet mass balance. The first, glacio-isostatic adjustment (GIA), is the solid-earth vertical response to deglaciation since the last glacial maximum ( 20k years ago). GIA results in the flow of heavy mantle material into the unloaded region, thus significantly affecting gravimetry readings. The second, firn compaction, is the mechanism by which snow at the surface compacts to become ice (generally over decades). Because this is a compaction process (i.e. a change in density but not in mass), it is not detected by gravimetry but by altimetry. Third, there is an elastic response of the bedrock to changes in ice loading. The elastic response and firn compaction are confounded, as both are not detected by gravimetry. However, firn compaction fluctuations are on the order of centimetre/year, whilst elastic responses on the order of a few millimetre/year. Because of this difference, we assume a zero elastic response: every change in height, which does not cause a mass change, is assumed to be by firn compaction.

**Fig 1:**
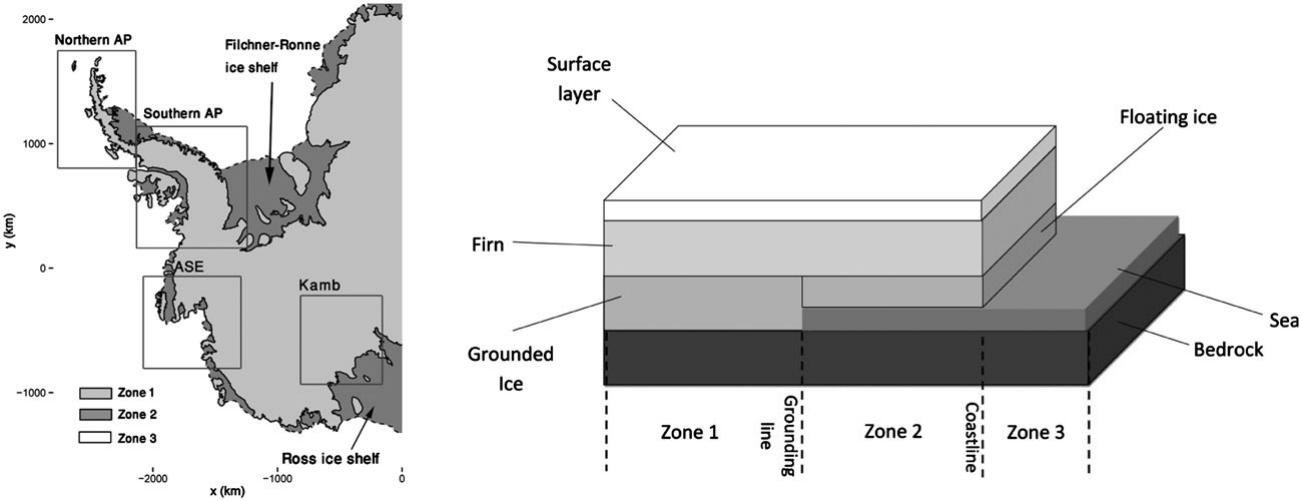
Left: The West Antarctic Ice Sheet (WAIS) and the AP with annotated regions of interest. The grounding line (-) marks the boundary of the grounded ice (Zone 1), whilst the coastline (- -) marks the ice-shelf (Zone 2) outline. All islands in the vicinity of the WAIS and the AP with an area less than 1000 km^2^ are omitted from the study. Right: The four layers (bedrock, ice, firn and surface) and the three observation zones in the ice sheet. Gravimetry cannot detect any mass change apart from glacio-isostatic adjustment (GIA) (due to bedrock uplift) in Zone 2. Over the sea (Zone 3), the altimetry observations are non-informative, whilst GIA is still detected by gravimetry. Most of Antarctica can be considered to be a Zone 1 region

There are thus four processes that need to be accounted for: (i) surface processes, (ii) firn densification, (iii) ice dynamics and (iv) GIA. Whilst the former three need to be modelled to abruptly terminate at the Antarctic coastline (they all relate to ice), GIA may be taking place out at sea. Further, for mass budget estimates, it is important to assess whether the former three are within the grounding line (the point at which ice begins to float on the ocean and loses contact with bedrock), or outside it. Any melt in parts of the ice sheet, which are *not* grounded (i.e. floating ice shelves), does not contribute to sea-level rise. Observation modelling, discussed in Section 4.2, needs to cater for this spatial dependence. The coastline and grounding line demarcations may be seen in Figure 1 (left) for the West Antarctic Ice Sheet (WAIS) and the Antarctic Peninsula (AP). The WAIS and AP constitute about a sixth of the entire ice sheet. Because this is the first study of its kind, we will restrict our attention to this area which, however, contains the strongest contributors to sea-level rise in Antarctica (Rignot *et al.* 2008).

The four processes take place in the physical layers idealised in Figure 1 (right). Even with the use of a third data type (Global Positioning System (GPS) measurements of vertical bedrock motion), a deterministic consideration of all four results in an ill-posed problem (three observations, four unknowns), compelling researchers to make various prior assumptions of variable validity. In the work of Riva *et al.* (2009), firn compaction is assumed to play no role in observed height trends. Further, at each point in space, mass loss is assumed to be a result of changes in either ice or surface processes, not both. Both assumptions are motivated by the fact that changes due to surface processes and firn compaction are expected to be dominated by those from ice in the critical areas. In the work of Luthcke *et al.* (2013), GIA is assumed to be known using numerical models. The mass balance is then obtained by deducting the contribution of GIA from the gravimetry observations. This is the most direct way of estimating ice sheet mass loss, but the modelled GIA correction is uncertain and various divergent estimates exist (Horwath and Dietrich, 2009). GIA has length scales on the order of hundreds to thousands of kilometres. Hence, errors (which would be spatially correlated) of even a few millimetre/year can induce considerable changes in mass budget estimates. Finally, Wingham *et al.* (2006) use only altimetry to estimate the ice mass loss. GIA and firn compaction are assumed to be of negligible relevance when compared to height change by ice loss and thus ignored. A regional climate model (downscaled global climate hindcast able to reconstruct surface processes) is then used to quantify the change in surface processes. Finally, the ice thickness anomalies are obtained after subtraction of the surface height change from the altimetry observations. This approach relies heavily on an accurate assessment of the surface processes by the regional climate model.

As discussed in the introduction, the hierarchical modelling approach presented here has several advantages over those discussed earlier. There is no requirement of applying numerical model outputs to physical processes and observation models may be constructed, which explicitly relate the measurements to the processes generating them. In addition, hierarchical modelling is a fully probabilistic Bayesian framework and is thus also ideally suited for quantifying uncertainty. Uncertainty in one of the latent processes (for example, the one that is least resolved in the data) is propagated naturally through all other processes. All these advantages are possible because hierarchical modelling is a *process-based* approach: whilst in the previous methods, prior process information (through numerical models or otherwise) is used to correct the data until they reflect the quantity being estimated (e.g. height change due to ice dynamics), with hierarchical modelling we start off by defining the characteristics of processes and then use the data to correct our prior belief. The investigated quantity is then obtained from our updated (posterior) understanding.

## 3. PROCESS MODEL

The height trends (in metre/year) of ice dynamics, surface and firn compaction are highly heterogeneous in space, especially along the coastline. The differing degree of heterogeneity amongst processes is key to separating the processes in the inference stage and thus must be captured in our prior model. Both (i) geostatistical (Cressie, 1993) and (ii) Gaussian Markov random field (GMRF; Rue and Held, 2005) approaches to constructing such models are easily suited to this task. The former is known to suffer from the *big n* problem because of the matrix inversion required for inference with a Gaussian field formulation. On the other hand, GMRFs are prone to loss of interpretability in the construction of the conditional-dependence matrix. Unlike Gaussian fields, GMRFs are, however, amenable to linear algebraic operations for fast, computationally tractable inference.

### 3.1. Stochastic partial differential equation formulation

As shown by Lindgren *et al.* (2011), the advantages of the two spatial modelling approaches may be reconciled by assuming that the random fields are Gaussian processes, which are solutions to heterogeneous stochastic partial differential equations (SPDEs) of the form 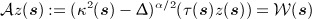
1 where 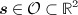 is the spatial domain of interest, *z*(***s***) is the height trend, Δ is the Laplacian, *κ*(***s***) > 0 is a length-scale parameter, *τ*(***s***) > 0 is a field-scaling parameter and 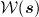 is Gaussian spatial white noise. The exponent *α* = *ν* + *d* / 2 where *ν* is a smoothness parameter and *d* = 2 is the spatial dimensionality. The length-scale parameter *κ*(***s***) is related empirically to a range parameter *l*(***s***) corresponding to correlations of 0.1 as 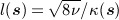.

The geostatistical interpretation of the random field is preserved in the SPDE formulation through Whittle's result (Whittle, 1954), which shows that for a spatially invariant (*τ*,*κ*), *z*(***s***) is a Gaussian field with a kernel of the Matérn-type. A local interpretation for heterogeneous fields follows naturally: for smoothly varying *κ*(***s***), *τ*(***s***) > 0, *locally* at *κ*(***s***) = κ* and *τ*(***s***) = τ*, *z*(***s***) is a Gaussian field with Matérn kernel 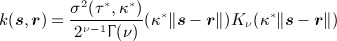
2 where *σ*^2^(τ*,κ*) is the marginal variance of the spatial process and *K*_*ν*_ is the modified Bessel function of the second kind. The field-scaling parameter *τ*(***s***) is related to the marginal variance of the SPDE 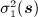 through 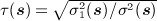. The computational advantages of a GMRF formulation are then taken advantage of through dimensionality reduction of the SPDE, see Section 3.2.

Each of the four processes, say *z*_*i*_(***s***), can be modelled through the SPDE formulation 1. The resulting spatial process is hence *multivariate*, that is, at each location ***s***, 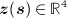 has four components, one for each process. Known dependencies between the processes introduce cross-covariance structures, which need to be included within the model (Gneiting *et al.* 2010).

#### The multivariate spatial process

Denote the height trends due to the four processes (bedrock, ice, firn and surface) as *z*_*R*_(***s***),*z*_*I*_(***s***),*z*_*F*_(***s***) and *z*_*S*_(***s***). The processes *z*_*R*_(***s***) and *z*_*i*_(***s***) are independent from each other and from *z*_*F*_(***s***),*z*_*S*_(***s***). The latter two are, however, correlated. Correlations arise from the fact that the rate of firn compaction is highly dependent on the precipitation rate: increased precipitation will cause increased compaction (Arthern *et al.* 2010).

Consider, for now, two dependent Gaussian processes *z*_1_(***s***),*z*_2_(***s***). Define the cross-covariance matrix function of ***z***(***s***) = [*z*_1_(***s***) *z*_2_(***s***)]^*T*^ as 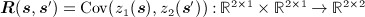. This may be constructed by modelling the joint field as a linear transform of two independent fields of unit variance 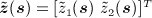, where Cov(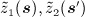) is diagonal, through a (possibly) spatially varying matrix transform 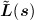


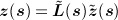
3 where 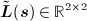 is the Cholesky factor of the cross-covariance matrix function ***R***(***s***,***s***) (Finley *et al.* 2007). In this application, we find no reason to model a spatially variant dependence between the two processes and thus enforce spatial invariance through setting ***R***(***s***,***s*** ′ ) = ***R***, giving 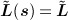. This is the simplest possible construction of covarying processes in what is commonly termed the coregionalisation approach (Wackernagel, 2003).

As a further simplification, suppose that *z*_1_(***s***) and *z*_2_(***s***) are a linear combination of two fields 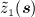 and 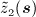, which are i.i.d. Then, without loss in generality, one may fix 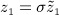 where *σ*^2^ is the marginal variance of *z*_1_ and model 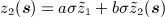 so that 
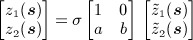
4 The (pointwise) cross-covariance matrix 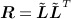 is then given by 
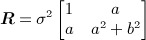
5 This special case is of interest to us as visual inspection of projected height trends in numerical models due to firn densification and surface process reveal practically identical spatial frequency characteristics. We use the similarity to justify the ‘identically distributed’ (up to some variance *σ*^2^) assumption. Let *z*_1_(***s***) = *z*_*S*_(***s***) and *z*_2_(***s***) = *z*_*F*_(***s***). An estimate for ***R*** is the empirical covariance matrix 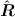, which we obtain using a regional climate model (Lenaerts *et al.* 2012) and a firn densification model (Ligtenberg *et al.* 2011): 
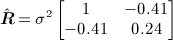
6 Equation 6 shows the negative correlation between the two processes and the smaller amplitude changes associated with trends in firn densification. The estimate 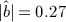 (using 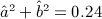) is smaller than 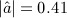 indicating that the trends due to firn processes are in fact strongly dependent on those at the surface.

#### System of SPDEs

The previous discussion leads us to the consideration of a system of SPDEs (Aune and Simpson, 2012). Consider once again 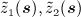. Because these are i.i.d., they may be represented as follows: 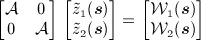
7 where 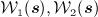 are i.i.d. Without loss of generality, we assume that *σ* = 1 and that the marginal variance is embedded in 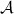. Then, by 4, we have that 
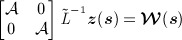
8 where 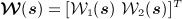. Equation 8 may also be given in Kronecker form by treating 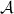 as a matrix of size 1 × 1 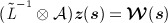
9 This formulation using the Kronecker product results in considerable computational simplifications, which will be taken advantage of in Section 3.2. We now construct the system governed by the problem under study. Denote the operators associated with the rock, ice, firn and surface processes as 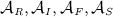, respectively. Then the modelled random fields are the solution to the system 
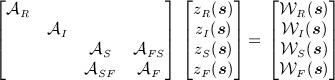
10 where the sub-block 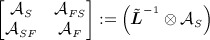
11 The solutions to 10 are 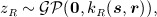
12a

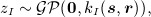
12b

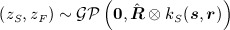
12c where 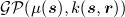 denotes a Gaussian process with mean *μ*(***s***) and covariance function *k*(***s***,***r***). The random fields (*z*_*S*_,*z*_*F*_) are jointly Gaussian with dependence defined through the cross-covariance matrix function.

#### Matérn configuration

The natural question to ask is what to choose for *τ*(***s***),*κ*(***s***) in 2, or, equivalently, *σ*(***s***) and *l*(***s***) (the marginal deviation and range parameter, respectively), which we assume are slowly varying. The parameters are needed to configure 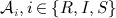
—note that because of the dependence in 9, we do not need to explicitly specify 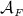. The marginal deviation and range parameters can be themselves decomposed into smooth basis functions whose weights are subsequently estimated in a parameter estimation step. However, we find that numerical models of ice sheet processes are ideally suited to set the local properties of the covariance function. The models incorporate knowledge of geophysical characteristics such as elevation and precipitation levels, which correctly capture the spatial variability (in terms of range and field-scaling). Further, the resolution of the numerical models is generally sufficiently high to extract the local characteristics.

**Fig 2:**
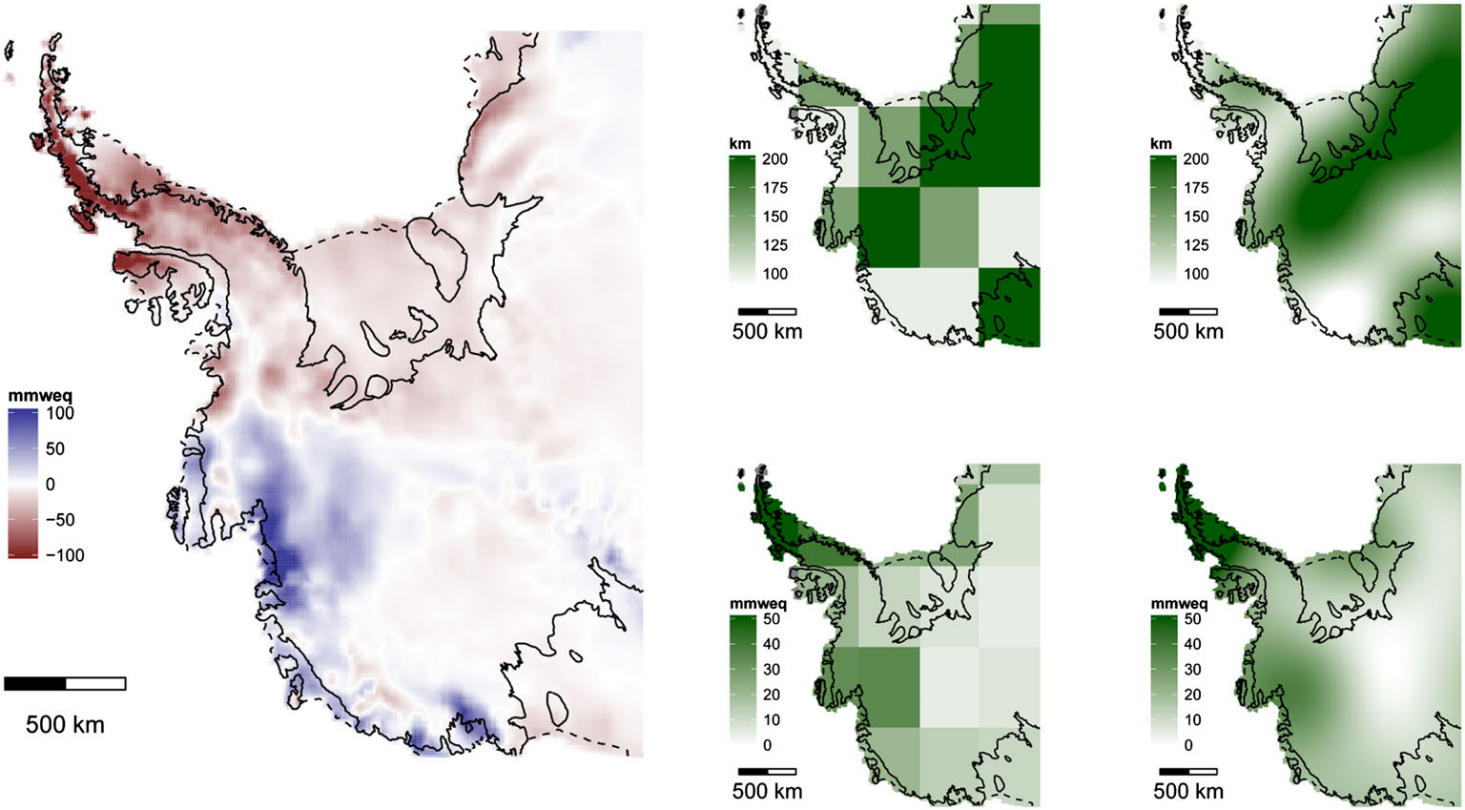
Left: Surface process anomalies in mm water equivalent (mmweq) as determined from the regional climate model. Right: Characteristics *l*(*s*) (top) and *σ*(*s*) (bottom) obtained by fitting local Matérn fields to the regional climate model anomaly on a grid (left sub-panels) subsequently smoothed out using Gaussian radial basis functions (right sub-panels)

The first numerical model outputs we consider are the surface process anomalies (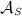), calculated as the difference between the study-period mean (2003–2009) and the long-term mean (1979–2002). The long-term mean varies from 4000 mm water equivalent (mmweq) at the coast to a few hundred mmweq further inland and is thus spatially heterogeneous. Note that a 10 mmweq anomaly results in a 10 mmweq/year trend in height change. We found *σ*(***s***) and *l*(***s***) using the model output in conjunction with fixed-subregion analysis (Haas, 1990). We divided the domain of interest into a 6 × 6 grid of squares with sides roughly 600 km in length and fitted zero-mean Matérn fields (*ν* = 3 / 2) to provide local estimates for l* and σ* collapsed to point estimates at the grid cells' centres (all distances and areas quoted in a polar stereographic projection at 71^*o*^ latitude). We next interpolated the points using Gaussian radial basis functions with standard deviations of 400 km (see also Higdon, 1998). The procedure for obtaining *l*(***s***) and *σ*(***s***) for surface process anomalies is shown in Figure 2. The result reflects our prior belief that surface processes close to the coast have a shorter wavelength and are larger in magnitude than in the drier interior. The same procedure was used for configuring 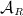. In this case, the GIA model we employed (Simon *et al.* 2010) generated a field, which is sufficiently regular and homogeneous to warrant spatially invariant *l* = 3000 km and *σ* = 4.2 mm. The parameters *σ* and *l* were found by fitting once again a zero-mean Matérn to the model output.

Finally, we need to configure 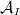. In this work, we do not make use of any numerical models, which predict change in height due to ice dynamics. Instead, we assume *a priori* a Matérn kernel with range parameter *l* = 50 km. This parameter is sufficiently large to accommodate regularity in large glaciers (e.g. the active Pine Island Glacier system) and sufficiently small for adjacent glaciers in the AP to be considered independent. However, we have a good indication of where mass loss, due to ice dynamics, is likely to occur by considering the ice surface velocity field obtained from satellite observations. In most cases, ice loss or gain can only occur where ice velocities are significantly high (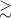
10 m/year) (e.g. Hurkmans *et al.*, 2012). We reflect this prior knowledge by setting the marginal standard deviation of the ice-loss trend to a sigmoid function: 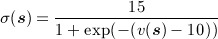
13 where *v*(***s***) is the ice velocity as detected by synthetic aperture radar (Rignot *et al.* 2011) and the saturation limit 15 m/year is a soft maximum for the ice-loss trend. One exception to this is the Kamb ice stream in the southern part of the WAIS. Here, ice accumulation is occurring at low ice velocities after the shut-down of a previously fast-flowing glacier, which occurred 150 years ago (Ng and Conway, 2004). We cater for this by setting *σ*(***s***) = 0.5 m/year in this drainage basin.

Combining physical model output and statistical models in this way was carried out for the first time, to the best of the authors' knowledge, by Calder *et al.* (2011). Similar to this work, they use the numerical model output solely to elicit the dynamic characteristics of a spatio-temporal model able to describe flow direction and dispersion. Another approach to physical-statistical modelling, not used here, is to use physical models to assist dimensionality reduction (e.g. Wikle *et al.*, 2001). Constraining modes of variation in a subset of the physical processes would also help in reducing posterior uncertainty on the marginal process distributions.

### 3.2. GMRF representation

In order to maintain a feasible computational complexity, we translate the Gaussian field representations into approximate GMRF representations (Lindgren *et al.* 2011). To do so, we discretise the system of SPDEs described in 10 using a basis of compact support. We construct finite elements on a triangulated domain in order to easily fit boundaries of arbitrary shapes and to accommodate a mesh density, which varies according to the spatial characteristics of the represented processes. We stress that in this case, the spatial properties are fully determined by the SPDEs and not by the triangulation/discretisation employed, which serve only to construct approximate solutions. This approach is hence different from standard GMRF modelling where the spatial characteristics are usually dependent on the specific discretisation employed.

**Fig 3:**
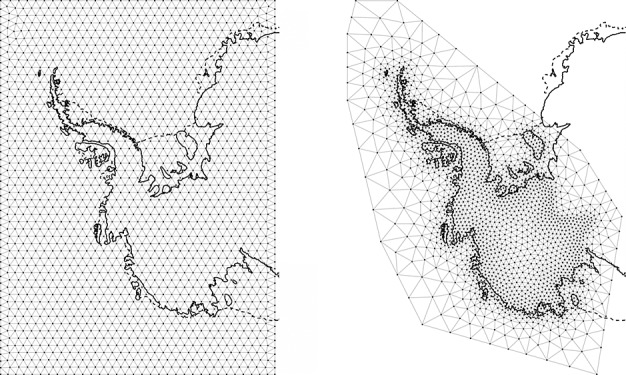
Left: Triangulation for discretising glacio-isostatic adjustment. Right: Triangulation for discretising ice/surface/firn processes. Although ice dynamics and surface processes occur in between the grounding line (-) and the coastline (- -) they are not important for mass budget studies and hence modelled at a lower resolution (see Section 3.2)

We generated triangulations from the algorithms of Persson and Strang (2004), which maximise the minimum angles of the triangles (*Delaunay* triangulations). Because no heterogeneity is apparent from the GIA numerical models, we generated a regular triangulation with a minimum triangle length of 100 km. Firn densification, surface and ice processes on the other hand, as shown previously, are heterogeneous in nature, with range parameters generally reducing with proximity to the coast. We hence used the following proxy for mesh density (passed as the scaled-edge length function) for the three processes 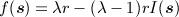
14 where *λ* > 1 penalises for whether the vertex lies within the mainland grounding line or an island (*I*(***s***) = 1) or not (*I*(***s***) = 0), and *r* is the distance from the region of interest (the WAIS + AP grounding line delineation and neighbouring islands with area larger than 1000 km ^2^). In this case, we chose *λ* = 8 and a minimum edge length of 40 km, which is sufficient to capture the majority of processes at the coast and small enough to encourage spatial regularity between neighbouring cells in the inferred ice process, see Figure 3. For all meshes, we used extended domains in order to reduce boundary effects. The total number of nodes in the mesh was *n* = 7972: 1744 for reconstructing the GIA and 2076 for the ice, firn and surface processes, respectively.

A set of basis functions on the constructed triangulation is found by setting each individual node to one and the rest to zero, for all nodes. The resulting basis functions, commonly known as *tent functions*, equal the field at their nodal points. Hence, for any decomposition 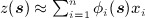, for each *ϕ*_*i*_ with central node ***s***_*i*_ *z*(***s***_*i*_) = *x*_*i*_. Moreover, the triangular graph is sparse: using the same graph to construct a GMRF prior will thus also result in sparse precision matrices.

We follow the approach of (Lindgren *et al.* 2011) and employ the Galerkin method by decomposing each underlying height trend using the set of basis functions 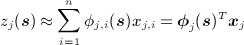
15 where throughout, *j* ∈ {*R*,*I*,*S*}is used to denote the rock, ice and surface fields, respectively. Each of these fields, governed by 1, is then projected through the inner product 〈 ·, · 〉with respect to ***ϕ***_*j*_(***s***) to give 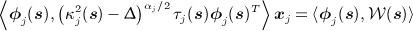
16 For *ν*_*j*_ = 1, *α*_*j*_ = 2, after application of Green's Theorem and zero Neumann boundary conditions, 18 reduces to 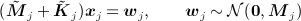
17 where 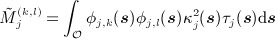
18

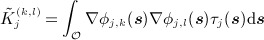
19

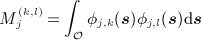
20

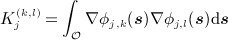
21 and the superscript (*i*,*j*) denotes the *i*,*j*^*th*^ matrix element. If *τ*_*j*_(***s***) and *κ*_*j*_(***s***) are sufficiently regular to be piecewise constant over 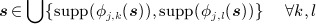
22 then 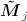 and 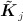 reduce to scaled versions of the mass and stiffness matrices ***M***_*j*_,***K***_*j*_, which can be obtained using standard finite element techniques. Because, from 19, 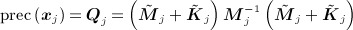
23 which is dense for non-diagonal ***M***_*j*_, we approximate ***M***_*j*_ as a diagonal using a row-sum lumping method (see also Bueche *et al.*, 2000). Note that from Lindgren *et al.* (2011) Section 2.3, *ν* (and hence *α*) are restricted to be positive integers in 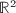. For GIA trends we employ *ν*_*G*_ = 2 (i.e. *α*_*G*_ = 3) fields. The discretisation of this smooth (homogeneous) field can be obtained using recursive Galerkin formulations, see Lindgren *et al.* (2011) for further details.

The reduction procedure results in a set of states for each process trend: ***x***_*R*_ for GIA, ***x***_*I*_ for ice dynamics, ***x***_*F*_ for firn compaction and ***x***_*S*_ for surface processes. The final concatenated state vector is 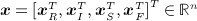, which is Gaussian with prior expectation ***x***^*p*^ = **0** and prior precision matrix 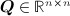, where ***Q*** is 4 × 4 in block form, with all four processes, except for ***x***_*S*_ and ***x***_*F*_, conditionally independent *a priori*. From 9, it follows that the finite-dimensional representation of the correlated fields is (recall that *σ* = 1 is assumed in 5) 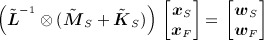
24 where ***w***_*S*_ and ***w***_*F*_ are i.i.d. with covariance matrix ***M***_*S*_. By repeated application of the mixed-product property of the Kronecker product, the block precision matrix is found as 
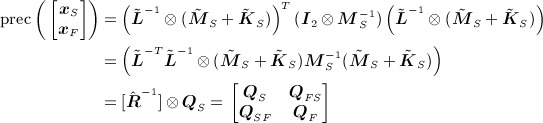
25 where ***I***_*m*_ is the *m* × *m* identity matrix. The full precision matrix is then similar in form to 10

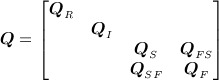
26 and is sparse. In the presence of known parameters, computation of the posterior distribution of ***x*** follows from the Cholesky factorisation; however, the nature of the observation model, described in the next section, hinders its direct computation for large (say 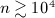) problems.

## 4. THE OBSERVATION MODEL

**Fig 4:**
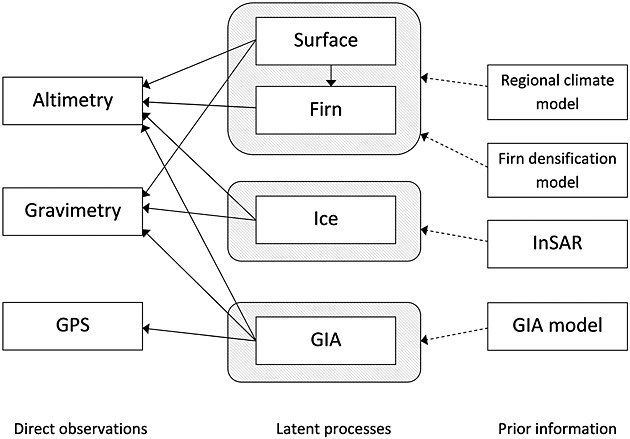
High-level information flow schematic. The altimetry, gravimetry and Global Positioning System (GPS) observations are generated (solid arrow) by the four latent processes, which contain prior information extracted from (dashed arrow) numerical models and ice velocity readings. The left and centre columns depict dependencies within the observation model, whilst the right and centre columns depict the information flow used for process model construction

We obtain spatially referenced temporal trend data using conventional trend analysis with ordinary least-squares by considering the largest time-window in which all data sets overlap: March 2003 to November 2009. With observations, the residuals are used to estimate trend errors (see, for example, Yan and Su, 2009). We make use of three data sets (summarised as temporal trends): (i) satellite altimetry, (ii) gravimetry and (iii) GPS. The fourth data set, synthetic aperture radar interferometry (InSAR), is used solely for prior construction. These are discussed in turn. Figure 4 depicts their scope in relation to the hierarchical model.

### 4.1. Data

#### 4.1.1. Altimetry

Elevation changes are key to estimating trends in mass loss. Two satellite altimetry data sets are used in this work: the Ice, Cloud and Land Elevation Satellite (ICESat), which is laser-based, and the Envisat, which is radar-based. For a comparison between the two, see Connor *et al.* (2009). Because the footprint on the ground (i.e. the area illuminated by the satellite instrument) of ICESat is 70 m and that of Envisat is 2–3 km, the two altimetry data sets are able to provide high-resolution detail in the resolved fields. ICESat was corrected for a bias of − 6.5 mm/year ± 2.9 mm/year, found through a comparison to (known) mean sea-level rise. ICESat and Envisat provide sufficient detail for temporal trends in height to be estimated at a 1 km resolution. However, it is likely that small-scale effects (Cressie and Wikle, 2011; Katzfuss and Cressie, 2012) and correlated errors (Wingham *et al.* 1998) highly corrupt height trends obtained using altimetry at this scale. Although these characteristics can be included into the observation model, for simplicity, we follow Riva *et al.* (2009) and assume that they can be ignored when averaging the 1 km trends on a 20 km grid. The standard deviation of the trends within every 20 km grid cell is then used as the aggregated error.

#### 4.1.2. Gravimetry

**Fig 5:**
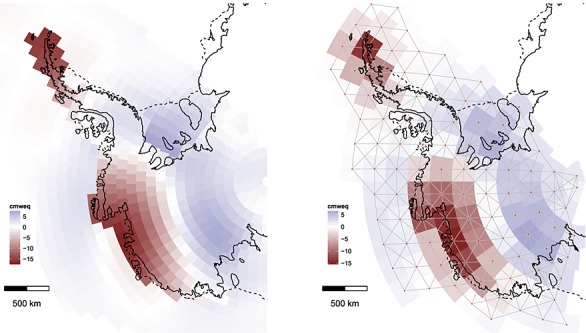
Left: Original gravimetry mascons. Note how these original readings are spatially smooth. Right: Aggregated mascons together with assumed neighbourhood structure of *P* (450 km local neighbourhood)

The Gravity Recovery and Climate Experiment records changes in the gravity field due to mass redistribution at and below the Earth surface and generates data, which may explicitly be used to quantify mass change. The data are made available in mass concentrations (mascons), which lie roughly on a 100 km polar grid. These, however, are an upsampling of a lower-frequency signal (Luthcke *et al.* 2013; Wahr *et al.* 1998). To simplify the highly correlated observation process, we averaged the signal on a polar grid (arc 300 km, radius 250 km) and further assumed that the resulting mass trends ***y***_*G*_ are a diffused image of the localised mass 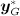, that is, 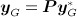 where 
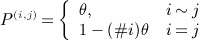
27 and where *#i* denotes the number of neighbours of mass concentration *i*, see Figure 5. A rough estimate for *θ*,*θ* = 0.1, was obtained by minimising 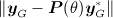 where 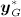 was simulated by using GIA and surface models and ice mass loss was estimated solely from altimetry readings. In the full inference scheme, *θ* is treated as an unknown parameter. Mascons were assumed to be neighbours in ***P*** if their geometric centres differed by no more than 450 km under the projection employed.

Signals obtained through gravimetry are compensated for atmospheric and ocean tide anomalies using forward models, which may contain systematic errors. In addition, there are leakage-in and leakage-out errors from unmodelled sources of mass variation surrounding the ice sheet (Luthcke *et al.* 2013). In this work, we thus assume that the true error of the gravimetry trend is a factor *γ*_*G*_ of the regression error where *γ*_*G*_ is treated as an unknown.

#### 4.1.3. Global Positioning System

Because GPS stations are placed on bedrock, GPS time series are able to provide an indication of the rate of GIA. Unfortunately, the elastic signal, caused by instantaneous changes in loading, is superimposed on that from GIA and can be larger in magnitude (Thomas *et al.* 2011). Unlike altimetry, the number of GPS readings is limited to a few dozen at most.

#### 4.1.4. Synthetic aperture radar interferometry

The InSAR (Rignot *et al.* 2011) is used to measure ice surface velocity. Unlike the other observations, the ice velocity map obtained from InSAR is solely used to help construct a spatially varying prior marginal variance for the ice field as seen in 15. The velocities in regions of the ice sheet not covered by the satellites or suffering high signal-to-noise ratio were replaced by balance velocities (i.e. the ice velocity required for an ice sheet in dynamic equilibrium) obtained from Bamber *et al.* (2000).

### 4.2. Observation zones

As in all geophysical applications, it is important to understand how the physical processes generate the data. A complication specific to this study is the spatial dependence of the observation process due to the distinction between the grounding line and the coastline. These spatial demarcations split up the domain of interest into observation zones (recall Figure 1).

Changes in mass of layers upstream (inland) of the grounding line (Zone 1) are detected by gravimetry. Floating ice shelves (Zone 2), which are downstream of the grounding line, are already in hydrostatic equilibrium, and melting in this zone does not contribute to sea-level rise. This spatial demarcation is important as it highlights from which regions, through the observational model, mass loss is permitted. The second demarcation, the coastline, separates the ice shelves (Zone 2) from the ocean (Zone 3). As stated previously, everything in Zone 2 is undetectable by gravimetry; however, height changes do occur and need to be incorporated in order to ensure smoothness of the surface, firn densification and ice processes across the grounding line.

These boundaries occur not only on mainland Antarctica but also on surrounding islands, all of which potentially also contribute to sea-level rise. Because grounding line/coastline data are not available other than for the mainland, we assume that all height changes within an island occur within the grounding line (Zone 1).

### 4.3. Model definition

Trends recorded by altimetry (averaged on a 20 km grid) are a superposition of the bedrock, ice, firn and surface height trends. For each altimeter recording 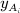 at spatial location ***s***_*i*_, the observation model is 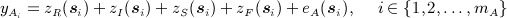
28 where *m*_*A*_ is the number of altimetry trends (on a 20 km grid) and 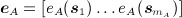 is a spatially uncorrelated noise vector independent of {*z*_*j*_(***s***_*i*_)}_*j* ∈ {*R*,*I*,*S*,*F*}_.

The observation model for GPS differs from that of altimetry because stations are situated on bedrock. For each GPS recorded trend 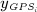 at spatial location ***s***_*i*_, the observation model is 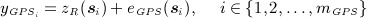
29 where *m*_*GPS*_ is the number of GPS stations. Equation 31 should also include the elastic response; however, this is of local scope and averages out over the large distances at which GPS stations are separated (generally several hundred kilometres). The elastic signal is thus assumed to be absorbed within the error term.

The gravimetry observations are slightly more involved as they record averages over an irregular domain with a spatial footprint of several thousands of squared kilometres. Each mascon reading is given as 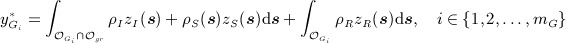
30 where *i* refers to the *i*^*th*^ observation, 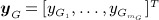 is the vector of gravimetry mass concentration trends, 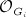 is the spatial footprint of the gravimetry reading, 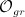 is the spatial domain enclosed by the *grounding line*, *ρ*_*I*_ is the density of ice (917 kg/m ^3^), *ρ*_*S*_(***s***) is the density at which surface process fluctuations occur (300–600 kg/m ^3^), obtained from the regional climate model, and *ρ*_*R*_ is the effective mantle density (3800 kg/m ^3^). Moreover, as noted earlier, the smoothing applied in post-processing results in the averages being a diffused version of the original reading themselves. Hence, from 29, the observation model becomes 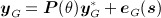
31 The error process 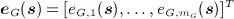 is assumed to be spatially uncorrelated because of the aggregation carried out (Section 4.1). To cater for temporal filtering, we set 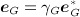 where 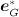 is the trend standard error. Note that 32 reflects gravimetry not detecting ice and surface mass change outside the grounding line. Also, because firn densification is a compaction process, it does not contribute to any change in mass and is hence not present in the observation model.

Finally, to enforce zero height trends over the sea due to surface, firn and ice processes outside of the coastline, we add pseudo-observations at all the relevant nodes. The model for pseudo-observations is 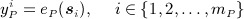
32 where var(*e*_*P*_(***s***_*i*_)) = 10^− 6^ introduces the confidence required on zero height trends. These pseudo-observations in Zone 3 are important to reconstruct the abrupt discontinuity present at the coastline. However, these are not critical for mass budget estimates as the demarcation is catered for during their calculation and also in the gravimetry observation process.

Following the Galerkin method on each *z*_*i*_(***s***), one can re-write the observation model in vector-matrix notation. Let 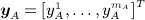 be the vector of altimetry trends, then ***y***_*A*_ = ***C***_*A*_***x*** + ***e***_*A*_ where 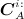, the *i*^*th*^ row of ***C***_*A*_, is given by 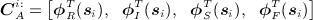
33 Similarly, let 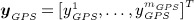 be the vector of GPS trends, then ***y***_*GPS*_ = ***C***_*GPS*_***x*** + ***e***_*GPS*_ where 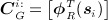. Pseudo-observations are treated similarly. Let 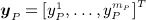, then ***y***_*P*_ = ***C***_*P*_***x*** + ***e***_*P*_ where 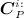 is zero everywhere except for the element corresponding to the node that is being softly constrained to zero; this element is set equal to 1.

For gravimetry, we first re-write 33 as follows: 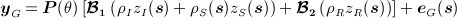
34 where 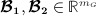 are vectors of linear operators, which compute the integral (to be numerically approximated) over the observational windows of the gravimetry observations corresponding to 32. The gravimetry observation model then reduces to ***y***_*G*_ = ***C***_*G*_(*θ*)***x*** + ***e***_*G*_ where ***C***_*G*_ = ***P***(*θ*)***D*** and 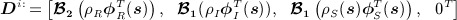
35

Define ***y*** = [***y***_*A*_,***y***_*GPS*_,***y***_*G*_,***y***_*P*_]^*T*^ as the observation vector and 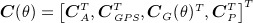 the respective observation matrix. In this problem, we have *m*_*A*_ = 8857, *m*_*GPS*_ = 22, *m*_*G*_ = 104 and *m*_*P*_ = 1483 so that 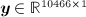. The full observation model can then be summarised as 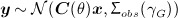
36 where Σ_*obs*_(*γ*_*G*_) is diagonal with all elements known (up to unknown 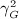) from the regression residuals. Note that because altimetry, GPS and pseudo-observations can be treated as point observations on a triangulated domain, each row of ***C***_*A*_ and ***C***_*GPS*_ contains at most three non-zero observations per process; these observation matrices are thus sparse. On the other hand, the sub-blocks corresponding to gravimetry, ***C***_*G*_, are dense, with the effect of correlating all the states in the update step, discussed next.

## 5. BAYESIAN ANALYSIS

**Fig 6:**
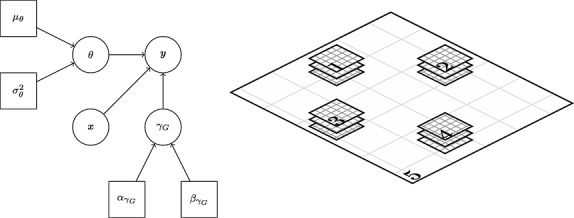
Left: Graphical model for Antarctic trend data. Right: Blocking (partitioning) the state-space to aid mixing. Ice, firn and surface processes are blocked together in small spatial groups of 600 km squares (Blocks 1–4). The glacio-isostatic adjustment field (Block 5) is grouped separately

The full model, graphically shown in Figure 6 (left), is as follows: 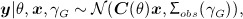
37a

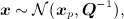
37b

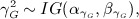
37c

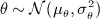
37d

A more suitable prior distribution for *θ* would be a beta distribution because 0 < *α* ≪ 1. However, the use of a normal prior distribution results in a normal conditional distribution, which can be readily sampled from in a Gibbs sampling step. Hyper-priors can form a fourth layer within the hierarchical framework; however, there is sufficient prior knowledge to treat these as fixed parameters. Following the study outlined in Section 4.1, we configured *p*(*θ*) to reflect our prior belief on the diffusion parameter of the gravimetry observations by setting *μ*_*θ*_ = 0.1 and *σ*_*θ*_ = 0.1. Finally, we reflect our uncertainty on the gravimetry variance-reduction by choosing 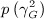 such that the 95 percentile *q*_0.95_ ≈ 400 and the 5 percentile *q*_0.05_ ≈ 1. Suitable values are 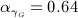 and the scale parameter 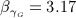.

For a given *γ*_*G*_ and *θ*, standard results (e.g. Rasmussen and Williams, 2006, Section 2.1) give 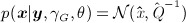 where 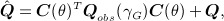
38a

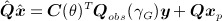
38b

Computation of the conditional posterior follows by finding the Cholesky factor of 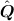. We then find 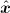 using standard linear algebraic routines. Computational efficiency relies on having a sparse Cholesky factor. However, for this problem, 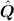 is relatively dense (7% full), which, by Rue and Held (2005) Corollary 2.2, implies a Cholesky factor of equal or higher density. One can reorder 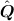 such that the number of non-zeros in the Cholesky factor is as low as possible. However, even with a suitable reordering, computation of the factor at each sampling step was considered prohibitive. This high density is attributed to the diffusion model for the gravimetry, which correlates all the states *a posteriori*. For illustration, if no diffusion was present (*θ* = 0), the density would have been less than 2%. For this reason, it is required to break-down the problem into smaller sub-problems. We thus partitioned the state-space into blocks from which Cholesky factors with moderate number of non-zeros could be computed. This allowed for quick and efficient sampling of the latent states.

When blocking, it is important to identify components, which, as much as possible, are loosely correlated in the stationary distribution. As the ice, firn and surface processes are considerably correlated *a posteriori* because of the superposition of the altimetry observations, we treated them simultaneously. However, because of their spatial roughness relative to the GIA signal, this subset of the state-space was divided into distinct spatial regions: poor mixing at the boundaries is expected to contribute little error to posterior inference. The GIA signal, due its large length scale, was treated as a single block. The latent field was thus partitioned as 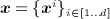 where *d* is the number of groups. A schematic of the blocking strategy is shown in Figure 6 (right).

Following the blocking, we generated samples using the usual deterministic sweep strategy (Roberts and Sahu, 2002) in which the sampler updates the individual blocks in consecutive order, always using the latest available update for the new sample. Once a sweep for ***x*** was completed, the parameters *θ* and *γ*_*G*_ were sampled from their conditionals, both of which are in the exponential family.

### 5.1. Initialisation

Irreducibility ensures convergence of a Markov chain within a Markov chain Monte Carlo framework to the stationary distribution. However, good starting values are able to reduce the burn-in period. This is useful when each sample is computationally expensive, as in the present case. A reasonable starting value can be obtained by computing the Cholesky factorisation of the posterior precision matrix for plug-in values of *θ* and *γ*_*G*_ in 2; however, this is not scalable with the current setup. For this reason, we approximate the Gaussian posterior using an approximate inference method.

Variational Bayes (VB) has been frequently employed in spatio-temporal modelling for fast approximate inference in applications where the likelihood is intractable (e.g. Zammit-Mangion *et al.*, 2012). However, VB can also be effectively used to approximate a tractable posterior by a simpler posterior, which is easier to compute, by taking advantage of the graphical model of the underlying latent field. Usually, this approximate posterior is one that assumes that subsets of the latent variables are independent conditioned on the data. Denote the indices of the *i*^*th*^ subset of ***x***, ***x***^*i*^, as 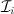 and define 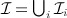. Then, for *d* subsets, the VB approximation is 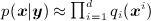. The partitioning scheme suggested previously is important in this case too as highly correlated blocks can lead to slow convergence and inaccurate approximate posteriors.

The approximate distribution in this problem is given as 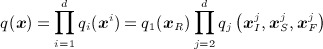
39 where *d* − 1 is the number of spatial partitions (in this case, *d* = 22). By the general theory of VB (Šmídl and Quinn, 2005), each approximate posterior is given by 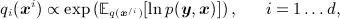
40 which, through conditioning, yields 
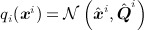
 where 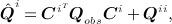
41a

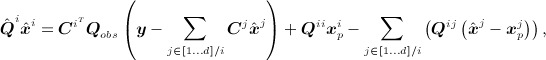
41b where 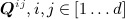 is the matrix composed of the rows in ***Q*** with indices in 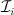 and the columns of ***Q*** with indices in 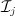, and where ***C***^*i*^ is the matrix composed of the columns in ***C*** with column indices in 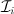. For a latent Gaussian process, 47 and 48 are identical to the Gibbs sampler conditionals with the expected means replaced with a sample value. Initially, we thought that sampling could be avoided and that VB could be used to obtain final estimates. However, careful inspection of the variational posterior estimates showed over- and under-confidence in some regions, prompting us to employ Gibbs sampling instead.

### 5.2. Gibbs sampler convergence

We ran the VB algorithm with plug-in values *θ* = 0.1 and *γ*_*G*_ = 10. The algorithm was assumed to have converged when the maximum change in the expectation of any state was less than 1 mm/year. Convergence was reached after 69 iterations and only required a few seconds using *R Software* (R Core Team, 2012) on a high-spec desktop computer. After setting the VB expectation as the first sample, we ran the Gibbs sampler for 5000 iterations and used the first 500 iterations as burn-in. Convergence of the samples was assessed through visual inspection and a Geweke diagnostic (Geweke, 1992) on the two parameters and a random subset of latent states (Wikle *et al.* 2001). The sampling procedure took 3 h to complete.

## 6. SUMMARY OF RESULTS

Despite the high-dimensional state-space inference is fast and produces promising results. In all figures, we use the triangulation's dual mesh, the Voronoi tessellation, in order to convey the resolution of the modelled fields.

### 6.1. Gravimetry parameter estimation

The results for the parameter estimates are given in Table 1. As expected, *θ* is centred at a reasonably high value (0.1) which, with the given neighbourhood structure, corresponds to an average signal diffusion of 60 *%*. This diffusion suggests a correlation length scale within the observations of several hundreds of kilometres and supports the evident smoothness apparent in Figure 5. The error amplification of 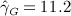 is a clear sign that trends estimated from the mascons are indeed over-confident. It is interesting to ask whether this high value renders the observations non-informative. Treating the mascon observation 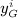 as the *signal*, we define the signal-to-noise ratio of each reading as 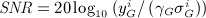. The gravimetry mascons have a maximum SNR of 37 dB with *γ*_*G*_ = 1, which drops to 16 dB at 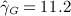. The latter corresponds to a reciprocal absolute coefficient of variation of 6 (i.e. the observed trend is six times its standard deviation) and can thus be considered informative.

**Table 1:** Parameter estimates for the gravimetry observation model

Parameter	5 percentile	50 percentile	95 percentile
*θ*	0.101	0.107	0.11
*γ*_*G*_	9.7	11.2	12.9

### 6.2. Process estimation

**Fig 7:**
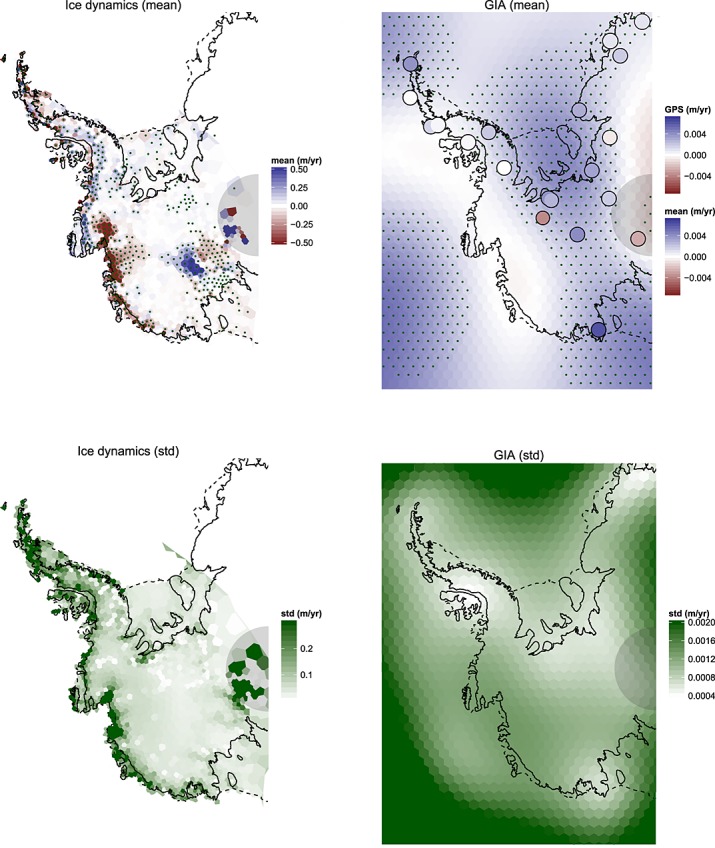
Left: Posterior expectation (top) and standard deviation (bottom) of height trends due to ice processes. Right: Posterior expectation (top) and standard deviation (bottom) of height trends due to glacio-isostatic adjustment (GIA). Stippling in the upper panels indicates an absolute posterior mean which is larger than the marginal standard deviation. Global Positioning System (GPS) readings are shown as points in the GIA mean field. The semicircle at the pole marks the region of no altimetry readings

**Fig 8:**
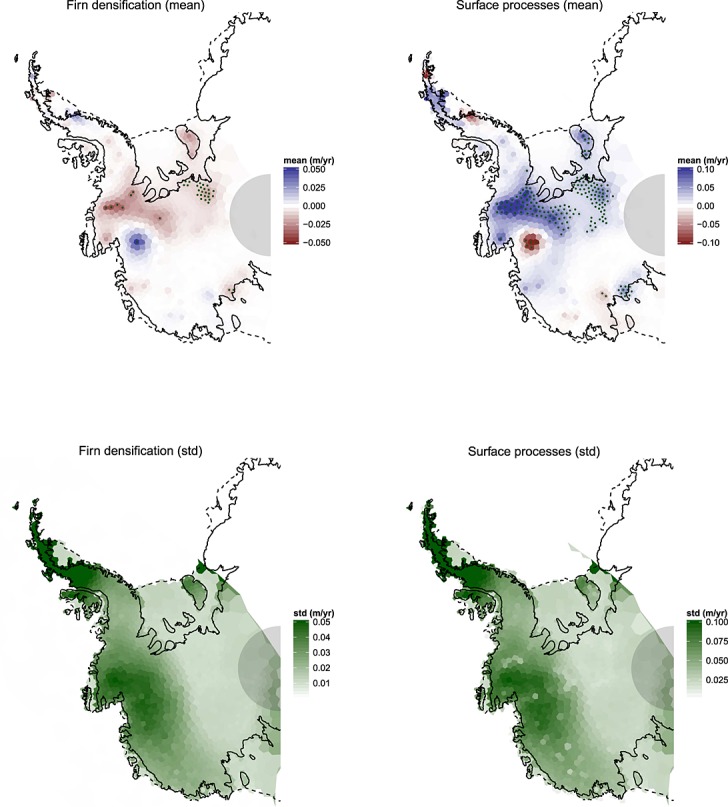
Left: Posterior mean (top) and standard deviation (bottom) of height trends due to firn densification. Right: Posterior mean (top) and standard deviation (bottom) of height trends due to surface processes. Stippling in the upper panels indicates an absolute posterior mean which is larger than the marginal standard deviation. The semicircle at the pole marks the region of no altimetry readings

In Figure 7 (left), we show the inference on the ice trend, accompanied by uncertainty estimates. Prominent features include the well-known ice-loss in the Amundsen Sea Embayment (Flament and Rémy, 2012) and ice accumulation in the Kamb ice stream (see Figure 1, left, for a map). Several isolated regions in the uncertainty map display high confidence. These correspond to regions of low ice velocity as recorded by InSAR and thus minimal height change due to ice dynamics. Height changes in these areas are attributed to surface and/or firn compaction processes, which in turn are inferred at lower uncertainty (Figure 8). In all trend maps, we show the circular region within which no altimetry readings are available. This is reflected in the ice process uncertainty map where regions of high velocity are also reported with high uncertainty.

Interestingly, considerable positive height change in the Southern AP is attributed to ice build-up, despite the generally accepted view that this region is losing mass overall (e.g. Kunz *et al.*, 2012). Trends in surface processes close to the coast cause mass loss at relatively high densities (close to that of ice), and as a consequence, there could be some confounding between ice and surface processes in this area. However, the regional climate model output shows a negative surface anomaly in this region (Figure 2, left). Moreover, there is some recent evidence from ice cores of a positive accumulation anomaly in the AP that predates the beginning of the regional climate model time series (1980). This corroborates our result (Nield *et al.* 2012; Kunz *et al.* 2012). Unlike in the Southern AP, ice loss and gain in the Northern AP display short wavelength variability. However, an overall negative trend in this region can be observed, which will be quantified explicitly in Section 6.3. The GIA trend in Figure 7 (right) is seen to be positive overall. This corroborates expert belief that there is an overall uplift of the bedrock in the WAIS. Peaks are evident over the Filchner-Ronne and Ross ice shelves, which is a common feature in forward numerical models. The peak uplift rate is 5 mm/year, which is about 2–3 mm/year less than that obtained from models (Sasgen *et al.* 2012; Whitehouse *et al.* 2012).

Most mass loss and height change is correctly attributed to ice dynamics, despite the ill-constrained nature of the problem. In the AP, where data are sparse, surface and firn process uncertainty is high. Firn densification is seen, by construction, to correlate negatively with the surface process. This is a realistic output but highly dominated by the prior information. A short discussion on this effect is given in Section 7.1.

The prior model-data balance is visualised by plotting the Kullback–Leibler divergence (KLD) between the posterior and the prior distributions at each point in space for each process. The KLD at some node 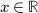 is defined as 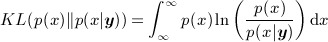
42 For computational reasons, we approximate the marginal posterior *p*(*x* | ***y***) as a normal density from the sample histogram. The KLD between two normals can be computed analytically, see for instance Roberts and Penny (2002), Appendix B.

**Fig 9:**
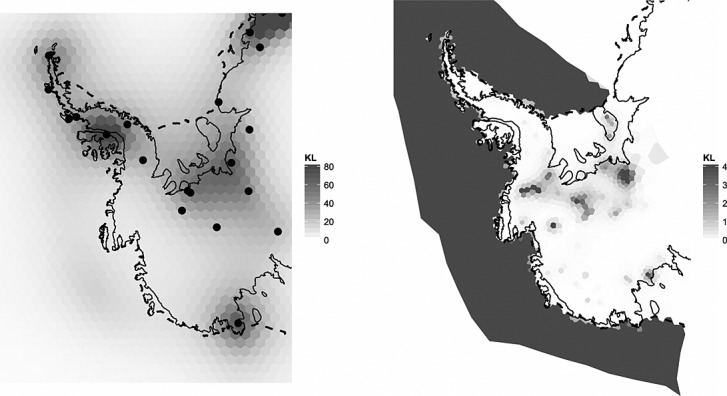
Left: Kullback–Leibler divergence (KLD) between the marginal prior and posterior states describing trends due to glacio-isostatic adjustment. Global Positioning System stations are denoted by dots. Right: KLD for surface (precipitation and ablation) processes

The KLD shows where data are significantly affecting our posterior estimates and, for this reason, is sometimes referred to as a measure of *surprise* (Itti and Baldi, 2006). Two representative maps are shown for the surface processes and GIA in Figure 9. A high KLD can indicate a shift in the mean, uncertainty or both. For GIA, we note that most adjustment occurs where GPS stations are relatively plentiful. The peak on the peninsula coincides with a GPS station, which provided an accurate trend estimate. KLDs of surface and firn processes are low over the peninsula where data are scarce. Unsurprisingly, adjustment for the surface trends is mostly present in regions of low ice velocity where detected height changes cannot be attributed to ice dynamics. High KLDs in Zone 3 are due to pseudo-observations (Section 4.3) forcing the posterior to zero beyond the coastline.

### 6.3. Mass-balance validation

We validate our approach on a sector level against estimates obtained using the input–output method (IOM, Rignot *et al.*, 2008) in 2006, half-way through our period of study. The IOM varies considerably to the approach taken here: whilst here, we estimate *trends* caused by a shift from a hypothetical balance state, the IOM simply subtracts an output (ice discharge) from an input (snow accumulation) in a given year. As a result, the role the data play in estimation is different. The IOM makes use of InSAR to directly estimate output flow, whilst in the present work, InSAR is only used to construct a prior on the magnitude of allowable height loss due to ice dynamics. One would thus expect the errors between the two to be largely uncorrelated, especially when considering the range of other data sources used here. Uncertainties in the IOM were estimated by comparison of the regional climate model and the firn densification model to *in situ* observations, and reported errors using InSAR (which, unlike here, play an important role in the estimated ice discharge).

**Fig 10:**
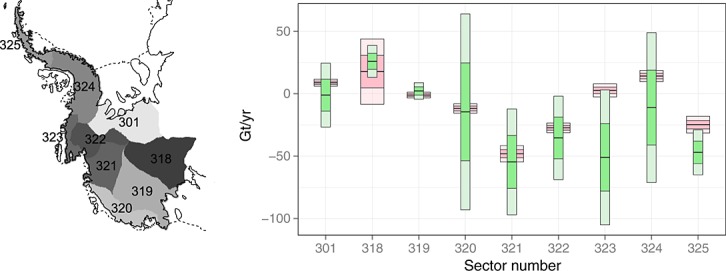
Left: Sector definitions. Right: Comparison between posterior estimates (pink, thick bars) and those obtained using the IO method in 2006 (green, thin bars). The 1 *σ* and 2 *σ* levels are denoted by the dark and light shadings, respectively

The results of the comparison are shown in Figure 10 and Table 2. The mean estimates are seen to mostly match within uncertainty levels. The large deviation of the means in Sector 324 is a result of the observed positive height trends being attributed to ice dynamics in the Southern AP. It is still unclear what is causing the large discrepancy in Sector 323, also apparent in the comparison by Shepherd *et al.* (2012), Figure 1. Our method is seen to report trends at a lower uncertainty. This, however, could be due to a number of factors. First, we are quoting uncertainty in trends, which span over 7 years (2003–2009), whilst the uncertainty of the IOM is for a single year. Averaging results obtained with the IOM over a similar time span would give lower errors (although the largest errors in the IOM are temporally correlated). Second, the model we employ still omits some sources of uncertainty concerning (i) the elastic response, (ii) the density profiles and (iii) the correlated errors within altimetry readings. Inclusion of these sources would increase our posterior uncertainty on all fields and hence also on our mass budget estimates.

**Table 2:** Posterior estimates from 2003 to 2009 trend data compared with the IO method in 2006

Sector	Estimated trend (Gt/year)	IO method (Gt, 2006)
301	8.80 ± 1.4	− 1.1 ± 12.8
318	17.7 ± 13.1	25.9 ± 6.4
319	− 1.1 ± 1.2	2.3 ± 3.3
320	− 11.8 ± 1.9	− 14.5 ± 39.2
321	− 48.1 ± 3.3	− 54.6 ± 21.2
322	− 27.3 ± 2.0	− 35.4 ± 16.7
323	2.7 ± 2.6	− 51 ± 27.0
324	14.2 ± 2.2	− 11.1 ± 30.0
325	− 24.8 ± 3.3	− 47 ± 9.0

Errors are at the 1 *σ* level.

### 6.4. Imbalance contribution to sea-level rise

We estimate that between 2003 and 2009, the combined mass loss rate of the WAIS and the AP was − 70 ± 15 Gt/year, which equates to a global sea-level rise of 0.20 ± 0.04 mm/year. Due to the mismatch in Sector 323, this is considerably lower than the IOM estimate of 0.52 ± 0.18 mm in 2006. Still, although this is a relatively short time interval and modest contribution to sea level, it represents about 10% of the mean rate for the 20th century. Importantly, the rate also appears to be accelerating (Rignot *et al.* 2011). This is of critical interest and importance as a large part of the WAIS is believed to be inherently unstable (Bamber *et al.* 2009; Schoof, 2007).

## 7. DISCUSSION

The major challenge in this field is the source separation problem. We briefly describe why this is the case in Section 7.1. In Section 7.2, we show how the problem features in some of the enhancements to the methods, which we are developing. Section 7.3 concludes the work.

### 7.1. Source separation

In signal processing, the separation of *source* signals from observed *mixtures* is known as blind source separation (e.g. Comon and Jutten, 2010). In the present case, the *sources* are the four physical processes, whilst the *mixtures* are the observed signals. *Blind* refers to the fact that the mixture process (analogous to the observation model in this context) is structurally unknown. Here, the observation model is structurally defined *a priori*; however, as we have more signals (four) than mixtures (three), the problem is under-determined.

Under-determined source separation has been recently applied in a Gaussian process context (Liutkus *et al.* 2011) for audio signal separation. Cardoso (1998) states that source separation is facilitated by *spectral diversity* and *mixture diversity*. Placing this into a BHM context, spectral diversity is manifested in prior process models having different covariance structures. For spatial processes, this translates to varying degrees of isotropy and/or length scales across the processes. The mixture diversity is reflected in the observation model, which is required to differentiate amongst the sources. Consider for example *x*_1_,*x*_2_ jointly Gaussian with zero mean. For an observation model *y* = *ax*_1_ + *bx*_2_ + *v* where *v* is independent from both *x*_1_ and *x*_2_, it can be easily shown that the posterior means 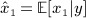 and 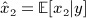 are linearly related through 
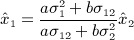
43 where 
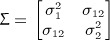
 is the posterior covariance matrix. This implies zero mixture diversity—the conditional mean of *x*_1_ is fully determined by that of *x*_2_ (and vice-versa), independent of the data. Although the conditional means might be separated, we term this type of separation *trivial separation*, as any separation is fully determined by the model specification and is data-invariant. One need not have perfect trivial separation in practice. A linear relationship between the posterior conditional means is, however, a strong indication of this effect.

Trivial separation is also possible in the spatial context. With no spectral dissociation (e.g. identical prior Matern fields), the posterior means of two Gaussian processes are perfectly linearly related under the observation model 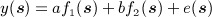
44 where *e*(***s***) is spatially uncorrelated. This is clearly seen in Figure 1 where the linear relationship diminishes in the case of prior spectral dissimilarity. However, neither differing prior marginal variances nor correlations between *f*_1_(***s***) and *f*_2_(***s***) remove this linear dependence.

**Fig 11:**
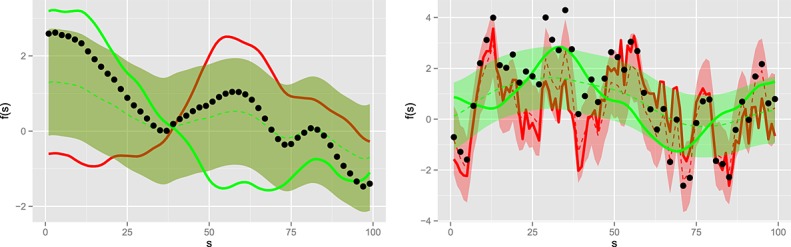
Impact of spectral diversity on source separation. The red and green lines indicate the signals to be separated; the dots are the observations following the observation model 51 with *a* = *b* = 1. The red and green dotted lines are the posterior means, whilst the ribbons denote the 1 *σ* interval (exactly overlapping in the left panel). Left: GP priors are Matérn fields with *ν* = 5 / 2. Right: Green signal prior is a *ν* = 5 / 2 Matérn, red signal prior is a *ν* = 1 / 2 Matérn

This scenario is of interest to us, as the firn process and surface process exhibit remarkably similar spectral properties. In fact, we equip them with identical (up to some linear correlation) process priors. Altimetry records a simple superposition of the four processes, which is hence only able to trivially separate the two processes. The separation can only be carried out using density differences through gravimetry. The diffused scope of gravimetry, however, makes this process extremely hard, and in fact, a near perfectly linear correlation is observed *a posteriori* between the surface and the firn conditional means. Trivial separation is not necessarily detrimental to the analysis; however, it does mean that caution should be exercised when expressing inferential statements—in this case, the function distribution of the firn process follows nearly deterministically from that of the surface process due to our prior assumed relationships between the two fields and not due to observed data. Unfortunately, the consideration of more processes, such as the elastic response, will only aggravate the source separation problem.

### 7.2. Framework extension

The simplest way to alleviate the source separation problem is to introduce more mixtures, that is, diverse data sets (increasing spatial coverage or accuracy of the individual instruments is less critical). In this work, we intentionally set up a framework that is able to readily incorporate data from other sources including, for example, those from *ice cores* (Frezzotti *et al.* 2012). Ice cores are obtained by extracting firn/ice samples from depths of a few tens to thousands of metres into the ice sheet, and give indications of annual snow accumulation layers over decades to millennia. Surface anomalies are relatively easily obtained from ice core data and could be treated as point observations (as with GPS), which could constrain the surface process layers. Indeed, this might be the only way to remedy trivial separability between the surface and the firn processes. In addition, spatially more extensive data relating to surface and firn processes are being generated from airborne surveys, which include the use of radar systems that can detect and track subsurface layers (isochrones) from the air. Such data are limited to relatively small sectors of the whole continent, but they are generally of high resolution and could also help further constrain and separate the latent fields, at least at a regional scale.

An important avenue for future work is the treatment of the temporal component within the process models. Spatio-temporal process priors may provide additional spectral dissimilarity required to separate the underlying physical processes. GIA, for example, is spatially and temporally smooth, whilst surface and firn processes have a high frequency temporal component (one day, for example, in the case of a snowfall event). Height loss due to ice dynamics is spatially rough but temporally rather smooth and without sub-annual variability. On the other hand, ice-shelf break-ups, such as that of Larsen B in 2002, result in sudden altimetric and gravimetric changes. These sudden change points provide a clear signature for ice dynamics. The spatio-temporal framework will have to allow for a spatio-temporal observation model, which would be achieved through the use of temporally indexed ***C***_*t*_. At a monthly resolution, computational complexity will have to be reduced by exploiting matrix sparsity. The use of *dynamic* spatio-temporal models in large-scale inference (as opposed to space-time Gaussian fields) is a complex problem and an active area of research (see, for example, Hartikainen *et al.*, 2011).

### 7.3. Concluding remarks

The analysis in Section 6 suggests that a statistical approach for determining the behaviour of the Antarctic ice sheet has a number of advantages over other methods that have been used in the past. The specific methodology designed for this work makes use of advanced statistical techniques that have revolutionised large-scale statistics in the last two decades. We do not claim that it is superior to those making more extensive use of numerical models, nor that the solution presented here is the final one. However, we present it as an entirely new way of approaching this challenging problem and as one that may open new doors in the analysis of ice sheet processes.

There are improvements that can be made to the current model. As mentioned in Section 6.3, the elastic response to ice loading, uncertainty in the densities *ρ*_*S*_(***s***) and *ρ*_*R*_, and the correlation errors within the altimetry may be modelled. All these modifications will invariably increase the posterior uncertainty. However, the optimal model was not meant to be the focus of the work, rather to introduce a new methodology. The tools we present here are versatile enough for the consideration of most stochastic models, which, in any case, may change as our understanding of processes in the cryosphere improves with time.
